# Next-generation, fast and accurate point-of-care test for NT-proBNP based on Magnotech technology

**DOI:** 10.1186/cc10791

**Published:** 2012-03-20

**Authors:** B Inçaurgarat, J Nieuwenhuis

**Affiliations:** 1 bioMérieux, Marcy L'Etoile, France; 2 Philips, Eindhoven, the Netherlands

## Introduction

In the emergency care setting, where time is of the essence, there is a need for fast and reliable information on NT-proBNP levels for diagnosis and management of acute dyspnea. Rapid NT-proBNP testing near the patient has the potential to streamline the process of care, but only if it is robust, fast and accurate enough to operate safely at the point of care (POC). Here we report on a novel NT-proBNP POC test which can be entirely carried out in a handheld device. This test has the potential to be rapid (<8 minutes), easy to use (fingerprick sampling), and with good accuracy compared to state-of-the-art automated laboratory assays.

## Methods

This new NT-proBNP POC test is based on Magnotech technology. A one-step sandwich immunoassay is performed in a compact plastic disposable cartridge with on-board dry reagents and magnetic nanoparticles. After a short incubation step the amount of bound nanoparticles, proportional to the concentration of NT-proBNP in the sample, is detected optically [1]. The precision of the assay was determined for plasma samples with NT-proBNP levels at clinically relevant values of 125 ng/l and 411 ng/l (10 replicates). Assay accuracy was determined by measuring 104 patient samples (lithium heparin plasma, NT-proBNP levels from 20 to 5,000 ng/l) on both the handheld device and the bioMérieux VIDAS laboratory system, and comparing results by Passing and Bablok regression analysis.

## Results

Assay precision was characterised by CV levels of less than 10%. NT-proBNP results correlated well with VIDAS (*r *= 0.89), with a corresponding slope of the regression line of 1.12 (95% CI 1.01 to 1.22) and an intercept of 64.04 (95% CI -73.50 to 109.83). In the current format under development, the NT-proBNP assay time with plasma samples is only 5 minutes. We are in the process of adding a filter that will allow measurements from whole blood directly. Flow experiments show that the filling time of the cartridge with whole blood is less than 30 seconds, resulting in a total assay time of less than 6 minutes, and a time-to-result of less than 8 minutes.

## Conclusion

In its current implementation the Magnotech-based NT-proBNP assay shows promising performance for rapid, reliable NT-proBNP testing at the POC in emergency settings. Development work is presently focused on the integration of a blood filter into the cartridge, to allow fingerprick tests.
